# Predictors of distant metastasis in parotid acinic cell carcinoma

**DOI:** 10.1186/s12885-019-5711-4

**Published:** 2019-05-21

**Authors:** Qigen Fang, Junfu Wu, Wei Du, Xu Zhang

**Affiliations:** Department of Head Neck and Thyroid, Affiliated Cancer Hospital of Zhengzhou University, Henan Cancer Hospital, Zhengzhou, Henan Province People’s Republic of China

**Keywords:** Parotid cancer, Intraparotid node metastasis, Acinic cell carcinoma, High-grade transformation, Distant metastasis

## Abstract

**Background:**

Distant metastasis (DM) is a common treatment failure pattern in acinic cell carcinoma (AciCC) of the major salivary glands; therefore, the main goal of this study was to analyse the predictors of DM in parotid AciCC.

**Methods:**

Consecutive patients with surgically treated parotid AciCC who were followed for at least 5 years were retrospectively reviewed. Data regarding age, sex, TNM stage, pathologic characteristics, surgical treatment, and follow-up examinations were collected and analysed. The primary end-point was DM control (DMC); the DMC survival was calculated from the date of surgery to the date of event or the latest follow-up examination and analysed by the Kaplan-Meier method. Independent prognostic factors were evaluated by the Cox proportional hazards method.

**Results:**

A total of 144 patients were included. Positive intraparotid nodes (IPNs) were noted in 34 (31.8%) patients. High-grade transformation was noted in 12 (8.3%) patients. A total of 83 (57.6%) patients underwent neck dissection, and neck node metastasis was proven in 37 (44.6%, 37/83) patients. The 10-year DMC rate was 86%. The Cox model analysis confirmed IPN metastasis (1.854 [1.061–4.144], *p* = 0.011) and high-grade transformation (4.219 [1.948–15.553], *p* < 0.001) as independent predictive factors of the DMC survival.

**Conclusion:**

IPN metastasis and high-grade transformation are independent prognostic factors of the DMC survival.

## Background

Acinic cell carcinoma (AciCC) of the major salivary glands is relatively uncommon, accounting for nearly 10% of salivary gland malignancies in China [1]. AciCC usually exhibits a relatively non-aggressive course [1–11]. Surgery is the most effective treatment, and adjuvant radiotherapy is suggested when there are adverse pathologic characteristics, including a high tumour stage, neck node metastasis, high-grade transformation, perineural invasion, and lymphovascular invasion [1, 3, 9, 11–14].

Although the reported prognosis and survival data in China are quite favourable, with a 10-year overall survival rate of > 80% [1], a number of patients suffer from disease recurrence. A study from the MD Anderson Cancer Center [9] reported that almost 20% of 155 patients with head and neck AciCC developed distant metastasis (DM), and both Gomez et al. [11] and Zeng et al. [6] reported that the most common treatment failure pattern was DM. However, none of these authors analysed the possible risk factors of DM. Although a number of clinical pathologic variables, including tumour stage, neck lymph node stage, disease stage and disease histology, have been reported to be associated with DM in salivary adenoid cystic carcinoma [15–17], whether there are similar phenomena in parotid AciCC remains unknown. Therefore, the current study aimed to evaluate the predictors of DM in parotid AciCC.

### Patients and methods

The Zhengzhou University institutional research committee approved our study, and all participants provided written informed consent for medical research prior to initial treatment. All experiments were performed in accordance with the relevant guidelines and regulations.

Consecutive patients surgically treated for primary parotid AciCC from January 2000 to January 2014 were retrospectively evaluated. Eligible patients were > 18 years of age, of either sex, able to provide written informed consent, and had been followed for at least 5 years. Data regarding age, sex, TNM stage, pathologic characteristics, surgical treatment, and follow-up examinations were collected. Intraparotid nodes (IPNs) are nodes located within the parotid tissue [18]. Postoperative radiotherapy is usually advised in our cancer centre when there are high-risk factors, including a high tumour stage, neck node metastasis, high-grade transformation, perineural invasion, lymphovascular invasion and IPN metastasis. After therapy, the patients were required to be examined every 3 months during the first year, every 6 months during the second year, and once per year after the second year by outpatient clinic visits, telephone calls, emails, or WeChat messages.

Systematic examinations were routinely performed for every patient via ultrasound, CT, MRI, and/or PET-CT. The TNM stage was formulated based on the 7th edition of the AJCC classification system.

The primary end-point was DM control (DMC), and the rate of survival with DMC was calculated from the date of surgery to the date of event or the latest follow-up examination. The Kaplan-Meier approach was used to calculate the rate of the DMC survival, and a multivariate Cox proportional hazards model was used to evaluate the independent prognostic factors. All statistical analyses were performed using SPSS 20.0, and *p* < 0.05 was considered significant.

## Results

A total of 144 patients (83 female and 61 male) were included in the analysis, and the mean age at diagnosis was 54.8 (range: 26–76) years. In 13 (9.0%) patients, the tumours were located in the deep lobe of the parotid. Total parotidectomy was performed in 105 (72.9%) patients, superficial parotidectomy was performed in 26 (18.1%) patients, and partial parotidectomy was performed in 13 (9.0%) patients. The pathologic tumour stages were distributed as follows: T1 in 42 (29.2%) cases; T2 in 61 (42.3%) cases; T3 in 27 (18.8%) cases; and T4 in 14 (9.7%) cases. A clear margin was achieved in 140 (97.2%) patients. High-grade transformation was noted in 12 (8.33%) patients. Perineural invasion was noted in 27 (18.8%) patients, and lymphovascular invasion was noted in 21 (14.6%) patients. Information regarding the IPNs was recorded in 107 (74.3%) patients; positive IPNs were noted in 34 (31.8%) patients (Table 1).

**Table 1:** Descriptive characteristics of the included patients

Characteristics	*N* (%)
Age	
< 55	74 (51.4%)
≥ 55	70 (48.6%)
Sex	
Male	61 (42.3%)
Female	83 (57.6%)
Tumor stage	
T1	42 (29.2%)
T2	61 (42.4%)
T3	27 (18.8%)
T4	14 (9.7%)
Neck node stage^a^	
N0	46 (55.4%)
N+	37 (44.6%)
Margin	
Clear	140 (97.2%)
Positive	4 (2.8%)
Tumour site	
Superficial	131 (91.0%)
Deep	13 (9.0%)
Perineural invasion	27 (18.8%)
Lymphovascular invasion	21 (14.6%)
High grade transformation	12 (8.3%)
IPN metastasis^b^	34 (31.8%)

^a^Only patients undergoing neck dissection were analyzed

^b^IPN: intraparotid node metastasis, only patients with IPN information were analyzed

A total of 31 (21.5%) patients were classified as cN+ and underwent therapeutic neck dissection (I-V); positive neck nodes were noted in 22 (71.0%, 22/31) patients. A total of 52 (36.1%) patients underwent prophylactic neck dissection (I-III) owing to adverse findings, including a high T stage and nerve invasion; positive neck nodes were noted in 15 (28.9%, 15/52) patients.

**Fig. 1:**
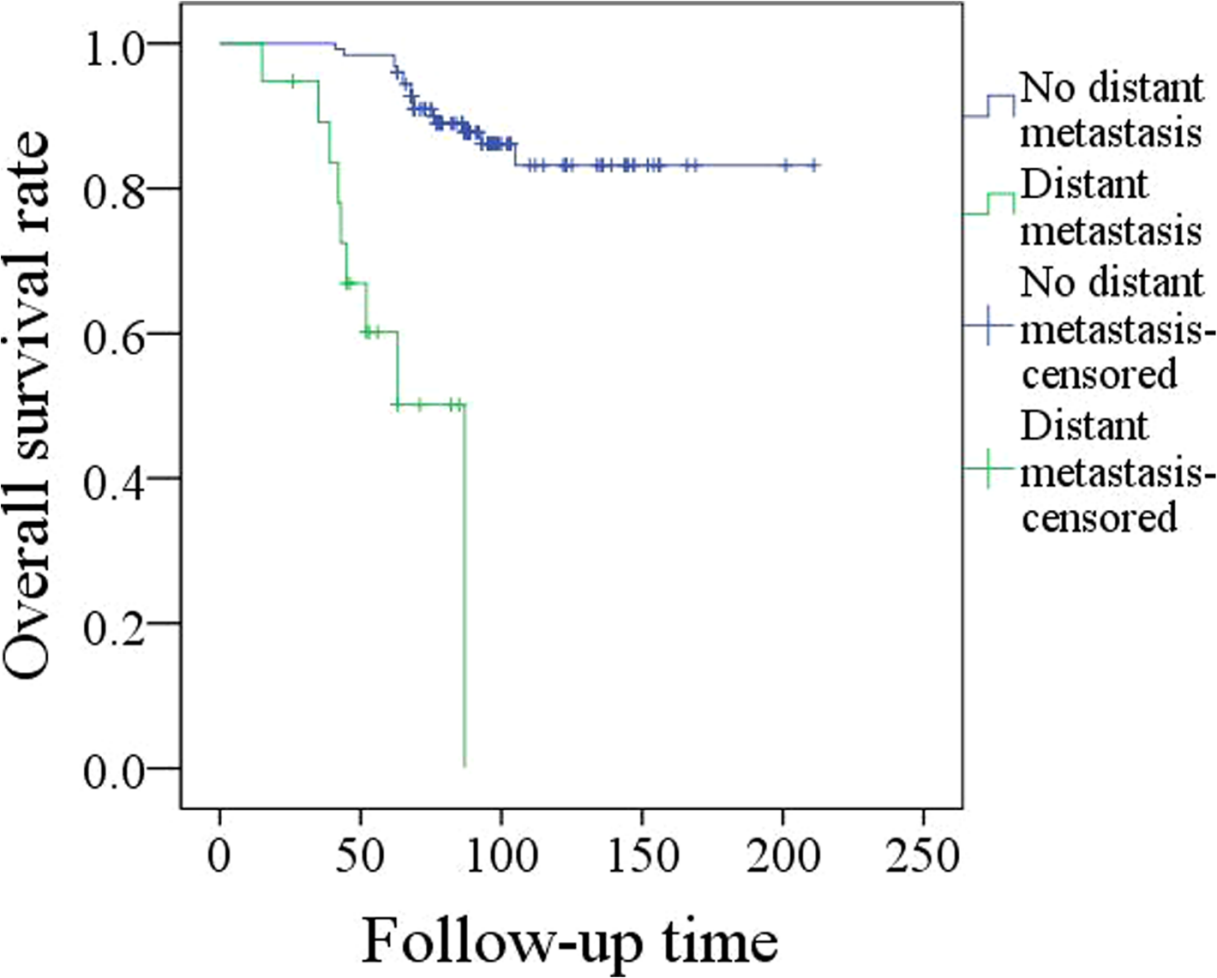
Overall survival in patients with and without distant metastasis (*p* < 0.001)

The mean follow-up time was 90.7 (range: 16–211) months. Adjuvant radiotherapy was performed in 48 (33.3%) patients, and 8 (5.6%) patients also underwent adjuvant chemotherapy. Recurrence was noted in 41 (28.5%) patients, and 19 (46.3%, 19/41) patients developed DM. When DM was detected, 8 patients had only DM, and 11 patients had simultaneous local or regional recurrence. Lung metastasis occurred in all 19 patients, liver metastasis occurred in 2 patients, vertebral metastasis occurred in 6 patients, sternal metastasis occurred in 2 patients, and rib metastasis occurred in 3 patients. The 5-year overall survival rates for patients with and without DM were 46 and 91%, respectively, the difference was significant (Fig. 1, *p* < 0.001).

**Fig. 2:**
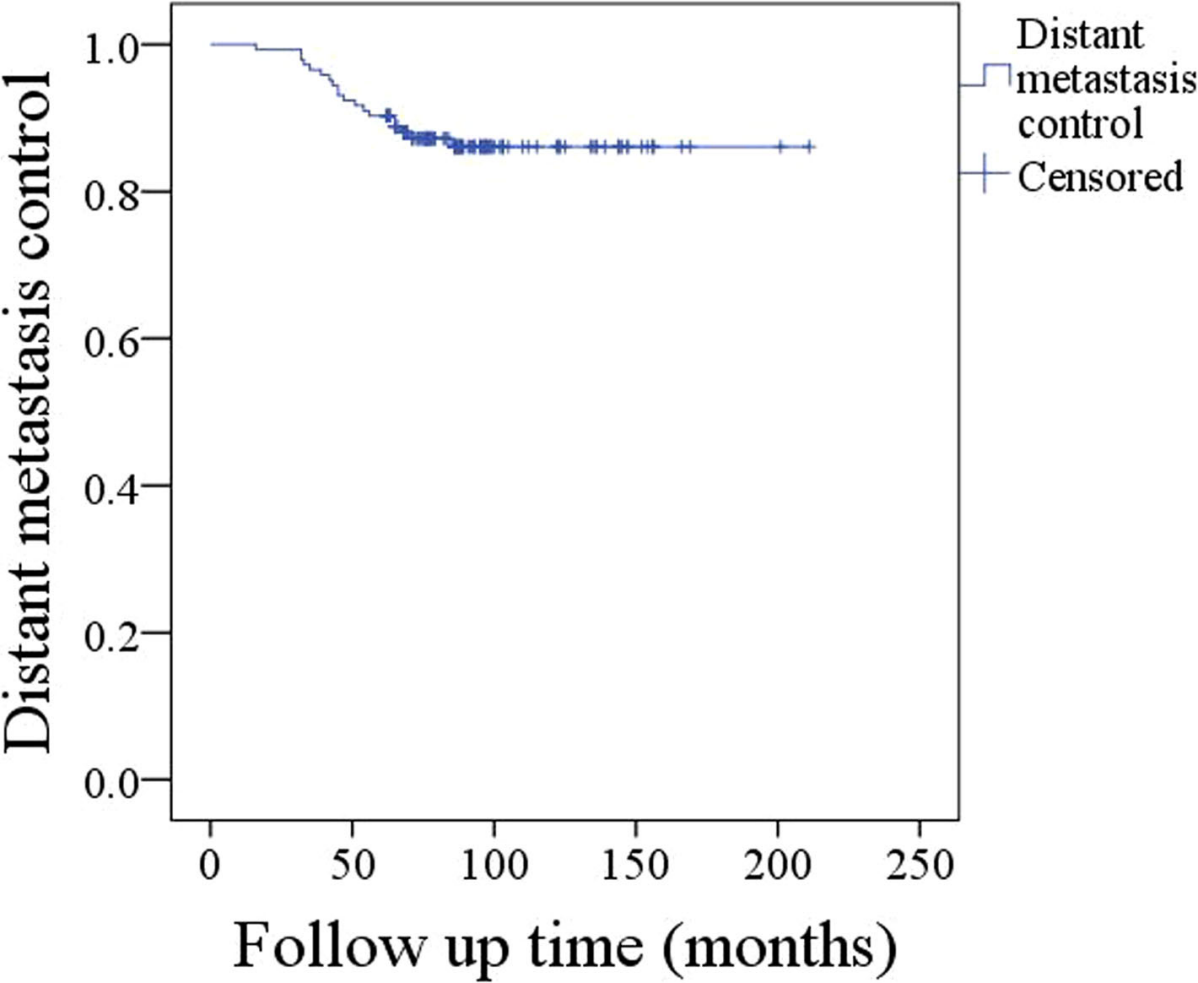
Survival with distant metastasis control in patients with parotid acinic cell carcinoma

The 10-year DMC rate was 86% (Fig. 2). Univariate analysis showed that age, sex, resection extent, tumour site, margin status, and perineural invasion were not significantly related to DMC (all *p* > 0.05), while a high tumour stage (*p* = 0.004), a high disease stage (*p* < 0.001), neck node metastasis (*p* = 0.011), IPN metastasis (*p* = 0.048), no radiotherapy (*p* = 0.035), high-grade transformation (*p* = 0.028), and lymphovascular invasion (*p* = 0.009) were associated with decreased survival with DMC. The Cox model analysis confirmed disease stage (*p* < 0.001, 3.874 [1.843–9.664]), radiotherapy (*p* = 0.005, 0.644 [0.433–0.893]), IPN metastasis (*p* = 0.011, 1.854 [1.061–4.144]), and high-grade transformation (*p* < 0.001, 4.219 [1.948–15.553]) as independent predictive factors of survival with DMC (Table 2).

**Table 2:** Univariate and cox model analysis of the predictors for distant metastasis control survival

Variables	Univariate	Cox model	
	Log rank test	HR (95% CI)	*p*
Age (< 55 vs ≥55)	0.258		
Sex (Male vs female)	0.371		
Tumor stage (T1 + T2 vs T3 + T4)	0.004	2.674(0.978–7.995)	0.089
Node stage (N0 vs N+)	0.011	3.612(0.287–13.611)	0.483
Disease stage (I + II vs III + IV)	< 0.001	3.874(1.843–9.664)	< 0.001
Resection extent (TP vs PP + SP)^a^	0.482		
Tumor site (Deep vs superficial)	0.337		
Margin (Positive vs negative)	0.687		
Perineural invasion	0.141		
Lymphovascular invasion	0.009	2.813(0.846–9.473)	0.219
High grade transformation	0.028	4.219(1.948–15.553)	< 0.001
IPN metastasis^b^	0.048	1.854(1.061–4.144)	0.011
Radiotherapy	0.035	0.644 (0.433–0.893)	0.005

^a^*TP* total parotidectomy, *PP* partial parotidectomy, *SP* superficial parotidectomy; ^b^*IPN* intraparotid node metastasis

## Discussion

DM is relatively uncommon in most head and neck malignancies; the reported incidence of DM in salivary gland cancers ranges from 20 to 50%, varying with different tumour sites and histologic types [14–17, 19]. The finding of the current study are consistent with those of previous reports. Although a few authors have presented the significance of several clinicopathologic characteristics, including cellular differentiation, inflammatory response, tumour invasion pattern, cartilage and/or bone infiltration, perineural infiltration, lymphatic and/or vascular invasion, capsular rupture, lymph node metastasis, and positive surgical margins, in predicting DM in salivary gland carcinomas [14–17, 19–21], no studies in the literature have evaluated the possible risk factors of DM in parotid AciCC. Parotid AciCC is known as a low-grade disease and usually has a good prognosis; therefore, parotid AciCC might have some unique predictors. The current study is the first to analyse the predictors of DM in parotid AciCC.

One of the main findings in the current study is that IPN metastasis apparently decreased the rate of the DMC survival. The value of IPN metastasis in parotid cancer has rarely been discussed. A few authors have reported that IPN metastasis is related to a high tumour stage, neck node metastasis, and adverse pathologic characteristics [13]. Moreover, Lim et al. [22] might have been the first to find that compared to patients without IPN metastasis, patients with a cN0 neck with IPN metastasis are more likely to develop locoregional recurrence. In a study published by Klussmann et al. [23], univariate analysis revealed IPN involvement as an additional significant risk factor of tumour recurrence in 55 patients with pN+ tumours. Nisa et al. [24] reported that decreased disease-free survival could be expected in patients with IPN involvement. Recently, we reported that IPN metastasis was associated with poorer local control and that patients with more than two metastatic nodes had the worst prognosis [25]. All of these findings suggest that IPN metastasis is related to a higher risk of recurrence. However, whether IPN metastasis increases the risk of DM remained unknown; we are the first to find that IPN metastasis carries a risk of nearly 2-fold for decreasing survival with DMC. The underlying mechanism might be as follows: first, the N parameter in the TNM classification refers to regional and cervical lymph nodes, and while IPNs appear to play a sentinel role in predicting neck disease, they are not included in any of the neck lymph node groups; second, IPNs include superficial and deep parotid nodes, and unresected positive IPNs might remain after partial or superficial parotidectomy [18].

Another interesting finding was that high-grade transformation predicted poorer DMC survival. High-grade transformation of conventional AciCC is uncommon and includes a variable proportion of a poorly differentiated high-grade component [3–5]. Transformed high-grade AciCC is related to poor clinical outcomes because of the tendency for recurrence and the common occurrence of angiolymphatic and perineural invasion. The clinical course in most cases is fatal, with dissemination followed by tumour-related death [3–5]. Similarly, in the current study, high-grade transformation appeared to have significance in predicting a poorer prognosis.

The role of adjuvant radiotherapy in increasing disease control in parotid cancer remains controversial. Zenga et al. [6] investigated whether there was some survival benefit associated with radiotherapy in parotid AciCC patients with close margins. The authors found that among patients without high-risk factors, those with close margins underwent significantly more radiotherapy, but this difference was not related to improved disease control. Andreoli et al. [8] evaluated the oncologic outcome of 1241 parotid AciCC patients and concluded that adjuvant radiotherapy did not seem to provide a significant survival advantage in early-stage or low-grade parotid AciCC. Similar findings have also been reported by Scherl et al. [3] and Neskey et al. [9]. However, Gomez et al. [11] examined overall survival and disease-free survival in patients with varying grades of AciCC that could be treated with adjuvant radiotherapy. Lin et al. [12] reported that radiotherapy could improve the long-term prognosis, especially in patients with advanced-stage disease or positive margins. In the current study, we found that the application of postoperative radiotherapy could improve the DMC survival. One possible explanation is that in our cancer centre, radiotherapy is usually suggested for patients with a high tumour stage, neck node metastasis, and adverse pathologic characteristics, among other features, i.e., those patients at a higher risk of DM.

Disease stage is known to be associated with prognosis in parotid cancer. The results of our previous studies indicated significantly decreased locoregional control and disease-specific survival in advanced-stage parotid cancer [13, 26]. Similar findings have been reported by Rajasekaran et al. [27] and Zhan et al. [28]. However, whether there is a similar trend regarding DM control remained unclear. Recently, Mariano et al. [14] established a relationship between a high disease stage and an increased risk of DM. In the current study, we also found that a high disease stage increased the risk of DM by nearly 4-fold, which confirms the importance of aggressive initial treatment [17].

Other factors, including age, sex, tumour stage, neck node stage, margin status, perineural invasion, and lymphovascular invasion, have been reported to be predictors of the prognosis in parotid cancer [2–11]. In a study consisting of 37 AciCC patients [2], the authors described their independent prognostic variables, including the clinical tumour stage, histopathology-related factors, and the pathologic nodal status. Neskey et al. [9] reported that the male sex, an age greater than 45 years and a tumour size larger than 3 cm were significantly related to decreased survival. However, the main end-point of these studies was survival with locoregional control, disease-specific survival or overall survival. In the current study, these factors were not identified as independent predictive factors of the DMC survival; thus, more studies are needed for clarification.

The limitations of the current study must be acknowledged. First, there is inherent bias in retrospective studies. Second, the sample size was relatively small, possibly reducing the statistical power; therefore, studies with larger sample sizes are needed to verify the results.

## Conclusions

In summary, a relatively favourable rate of long-term DMC survival was found in parotid AciCC patients, and IPN metastasis and high-grade transformation significantly decreased the rate of survival with DMC.

## Abbreviations

AciCC: Acinic cell carcinoma
DM: Distant metastasis
DMC: Distant metastasis control
IPN: Intraparotid node
